# On the occasion of world kidney day 2016; work together to better protect the kidney

**DOI:** 10.15171/jnp.2016.03

**Published:** 2015-04-20

**Authors:** Hamid Nasri, Mahmoud Rafieian-Kopaei

**Affiliations:** ^1^Department of Nephrology, Isfahan University of Medical Sciences, Isfahan, Iran; ^2^Medical Plants Research Center, Shahrekord University of Medical sciences, Shahrekord, Iran

**Keywords:** Chronic renal failure, World kidney day, Acute kidney injury, Renoprotection

## Abstract

*Context:* World kidney day is a yearly global alertness and education ceremony, held on the second Thursday in March.

*Evidence Acquisition:* Directory of open access journals (DOAJ), EMBASE, Google Scholar, PubMed, EBSCO, and Web of Science have been searched.

*Results:* Once again we reached to March 14, the world kidney day of 2016. This is the 10th anniversary of world kidney day, a program of the International Society of Nephrology (ISN) and the International Federation of Kidney Foundations (IFKF). World kidney day first began in 2006 and the worldwide campaign highlights a specific theme each year. The theme for 2015 was to invite everybody to drink a glass of water and give one, too, to celebrate their kidneys. This is a symbolic action to memorize that kidneys are vital organs and that they might be cared.

*Conclusions:* It is a manner to make individuals more conscious about their lifestyle choices. In this year, world kidney day will be celebrated on Thursday March 10, 2016. The theme for 2016 will highlight on renal disease and children.

**Implication for health policy/practice/research/medical education:**

World kidney day is a yearly global alertness and education ceremony, held on the second Thursday in March. In 2016, world kidney day will be celebrated on Thursday March 10, 2016. The theme for 2016 will highlight on renal disease and children. The general message of world kidney day 2016 is "a collaborative modality against the renal diseases in children that lead to end-stage renal failure, through increasing community outreach, improved economic opportunity, better education, and having access to preventive strategies for individuals at highest risk." These could end the unfavorable relationship between chronic renal failure and high risk populations.

## 1. Context

World kidney day is a yearly global alertness and education ceremony, held on the second Thursday in March. Once again we reached to March, the world kidney day of 2016 (1-3). In this year, world kidney day will be celebrated on Thursday March 10, 2016. The theme for 2016 will highlight on renal disease and children (2-4).

This is the 10th anniversary of world kidney day, a program of the International Society of Nephrology (ISN) and the International Federation of Kidney Foundations (IFKF). World kidney day first began in 2006 and the worldwide campaign highlights a specific theme each year, and the theme for 2015 is to invite everybody to drink a glass of water and give one, too, to celebrate their kidneys. In fact, this is a symbolic action to memorize that kidneys are vital organs and that they might be cared. It is a manner to make individuals more conscious about their lifestyle choices (3-8).

## 2. Evidence Acquisition

For this review, we used a variety of sources by searching through PubMed/Medline, Scopus, EMBASE, EBSCO and directory of open access journals (DOAJ). The search was conducted, using combinations of the following key words and or their equivalents; chronic renal failure, world kidney day, acute kidney injury and renoprotection.

## 3. Results

### 3.1. The history of world kidney day

From its beginning in 2006, world kidney day has become the most successful attempt to raise alertness amongst the general public and decision-makers about the significance of renal disease. Every year world kidney day reminds us that renal disease is common, harmful and also treatable (1-4). In this day, national and countless local events are organized by renal charities, healthcare professionals, patient groups and persons who wish to make a difference. We are encouraging everybody to get behind the campaign and endeavor to organize an activity or event to celebrate world kidney day and draw public notice to the need for renal healthiness, what can be done to save kidneys before disease strikes, and to help patients who previously have chronic renal failure. In fact, this day, provides the ideal opportunity to get out in the community and distribute the world kidney day message with family, friends and population you meet (2-5). In 2013, the theme of world kidney day was to increase awareness on acute kidney injury in both developed and developing countries (5-8).

### 3.2. Acute kidney injury may progress to chronic kidney disease

Acute kidney injury is defined by sudden decrease in kidney function through diminution of glomerular filtration rate, resulting in accumulation of nitrogenous waste products and electrolyte and fluid imbalance (8-10). This situation is frequently accompanied by decreased urine output and is highly associated with increased early and long-term mortality and morbidity. Notably also, there is consequently a risk for the progression to chronic kidney disease. In 2014, the aim of world kidney day was to increase awareness on chronic renal failure and aging (2-5). Chronic renal failure is characterized by slow loss of kidney function over time (7-9). Over the last decade, awareness on the incidence, rate of progression, risk factors and clinical outcomes of chronic renal failure has been growing as a result of high prevalence and rising awareness of chronic renal failure (3-9). In early stages of chronic kidney disease (CKD), patients are frequently asymptomatic (2-7). Although it could be asymptomatic, chronic renal failure negatively affects several organs, raises the risk of cardiovascular diseases and can progress to end-stage renal disease, and eventually increases the risk of hospitalization and death (9-12). There is an increasing prevalence of end-stage renal disease globally (11,12). The increase in prevalence of end-stage renal failure continues despite at least two decades of intensified renal protection programs including optimal blood pressure control, suitable glycemic control in diabetic patients, smoking cessation (5-9), and the broad use of angiotensin converting enzyme inhibitors or renin-angiotensin-aldosterone system blockers in both non-diabetic and diabetic chronic renal failure (9-14).

It is noteworthy to remember that the incidence of acute kidney injury and CKD has been growing over time. Though acute kidney injury has long been thought to be completely reversible disease, but, over the past several years, various data from experimental investigations and human studies have been published and pointed out that acute kidney injury can result in permanent kidney damage and progress to CKD (12-16). Additionally, the chance of patient survival after acute kidney injury has also been increasing over time (3,6-9). Hence, if acute kidney injury actually increases the risk for CKD, then it could delineate a noteworthy public health concern with attention to the percentage of individuals developing incident CKD, and end-stage kidney failure (14-19). The existing thought is that chronic renal disease will progress to end-stage renal disease or death at an increasing rate and progression from renal parenchymal damage to end-stage renal failure is the final common way for chronic nephropathies, which seems to be mainly independent of the initial kidney insult (14-19).

### 3.3. The concept of renoprotection

Each kidney has about one million tiny filters, called nephrons. If nephrons are injured, they will stop working. For an instance, healthy nephrons can take on the extra work. However, if the injury continues, more and more nephrons will be destroyed. After a certain point, the nephrons that are left may not filter the blood toxins well enough to maintain the homeostasis (12-19). At this time, there is an increased glomerular capillary pressure, which disturbs glomerular impermeability to proteins and lets an excessive quantity of protein to reach the renal proximal tubules. Subsequently, tubular reabsorption of filtered protein, results to renal interstitial injury by activating intracellular mechanisms, including up-regulation of the genes encoding vasoactive and several inflammatory mediators. At this stage, chronic renal failure as a progressive loss of kidney function over a period of months to years will ensue (8-14). However, various modalities have been proposed to delay the progression of renal failure. Renal interstitial inflammation and progression of disease can be delayed by angiotensin receptor blockade or angiotensin-converting enzyme inhibition which intensify the glomerular impermeability barrier to proteins and hence limit proteinuria and the filtered protein-dependent various inflammatory signals (7-15). Tubules can be protected by various natural or synthetic antioxidants against reactive oxygen species and various injurious insults which may result in tubular cell apoptosis and death and subsequent aggravation of renal failure (14-18).

Recently, much attention has been directed toward the concept of renoprotection by pursuing the above mentioned modalities to delay the progression of CKD. Fortunately, several clinical and experimental observations brightly imply that remission can now be achieved in some patients with chronic renal failure (18-24). It is clear that the proximal tubular epithelial cell is able to have a pro-fibrotic and pro-inflammatory role during proteinuria in which the proximal renal tubular epithelial cell expresses several chemokines and injury signals which result in extensive renal tubular atrophy, interstitial inflammation and eventually fibrosis (17-25).

More recently several studies have detected that chronic renal failure is a common condition which stimulates the cellular senescence and premature aging via toxic alterations in the internal milieu. This happens by some mechanisms, including DNA and mitochondrial injury, stem cell exhaustion, increased reactive oxygen species generation, phosphate toxicity and persistent inflammation, as well as telomere attrition. Moreover, in the process of various nephropathies, capillary rarefaction leads to local renal ischemia with further injury to the renal tubules, matrix protein deposition, presence of more profibrogenic mediators, fibrosis, and finally worsening the glomerulosclerosis (19-25). Therefore, in chronic renal failure, the tubules show various changes which are usually correlated with glomerular alterations, tubular cell degeneration, tubular apoptosis, and finally tubular atrophy (18-22). Therefore, it is reasonable to presume that we should provide tubular cell protection by using effective antioxidants (20-26). Moreover, administration of angiotensin converting enzyme inhibitors or renin-angiotensin-aldosterone system blockades or even a combination of them in appropriate patients in both non-diabetic and diabetic CKD. Additionally, control of blood glucose in diabetes and high blood pressure in hypertensive individuals is of utmost importance. These two diseases are the most leading causes of chronic renal failure and their incidence is growing at an alarming rate (23-26).

One of the important missions of world kidney day 2015 was to increase awareness on CKD in disadvantaged populations. Indeed, the increased burden of chronic renal failure in deprived populations is due to both universal factors and population specific issues (1-4). Low socioeconomic condition and poor access to care attributed to health care inequalities aggravate the negative impacts of genetic or biologic predisposition. Provision of suitable kidney care to these peoples requires providing facilities to the reach of dialysis by development of various low-cost alternatives and also providing facilities for renal transplantation (1-7).

### 3.4. The mission of world kidney day over years

The mission of world kidney day is to grow the awareness related to the kidney so that everyone cares for their kidneys and, if appropriate, have check-up to examine whether they are at risk for renal disease. Prevention of renal disease, early recognition, and subsequent renal protection are critical purposes for world kidney day (1-4). On the other hand, the global population is aging, and the number of persons above 85 years of age is developing faster than any other age group (3-8). As the population continues to age, physicians receive growing number of older patients with chronic renal failure and therefore, kidney failure would be a global challenge as a non-communicable epidemic (1-8).

## 4. Conclusions

The general message of world kidney day 2016 is “a collaborative modality against the renal diseases in children that lead to end-stage renal failure, through increasing community outreach, improved economic opportunity, better education, and having access to preventive strategies for individuals at highest risk.” These could end the unfavorable relationship between chronic renal failure and high risk populations.

## Authors’ contribution

Primary draft by HN. Editing the final manuscript by MRK.

## Conflicts of interest

The authors declared no competing interests.

## Funding/Support

None.

## References

[1] Tamadon MR, Ardalan MR, Nasri H (2013). World Kidney Day 2013; acute renal injury; a global health warning. J Parathyr Dis.

[2] Koyner JL, Cerdá J, Goldstein SL, Jaber BL, Liu KD, Shea JA (2014). The daily burden of acute kidney injury: a survey of US nephrologists on World Kidney Day. Am J Kidney Dis.

[3] Nasri H (2014). The awareness of chronic kidney disease and aging; the focus of world kidney day in 2014. J Nephropharmacol.

[4] World kidney day website. http://www.worldkidneyday.org.

[5] Tonelli M, Riellae M (2014). Chronic kidney Disease and the Aging Population. Arab J Nephrol Transplant.

[6] Hajivandi A, Amiri M (2014). World Kidney Day 2014: Kidney disease and elderly. J Parathyr Dis.

[7] Garcia-Garcia G, Jha V (2015). Environmental and occupational factors in CKD. Occup Environ Med.

[8] Tamadon MR, Zahmatkesh M (2015). World kidney day 2015. J Parathyr Dis.

[9] Drenth-van Maanen AC, Jansen PA, Proost JH, Egberts TC, van Zuilen AD, van der Stap D (2013). Renal function assessment in older adults. Br J Clin Pharmacol.

[10] Dhaun N, Webb DJ (2013). The road from AKI to CKD: the role of endothelin. Kidney Int.

[11] Go AS, Chertow GM, Fan D, McCulloch CE, Hsu CY (2004). Chronic kidney disease and the risks of death, cardiovascular events, and hospitalization. N Engl J Med.

[12] O’Hare AM, Choi AI, Bertenthal D, Bacchetti P, Garg AX, Kaufman JS (2007). Age affects outcomes in chronic kidney disease. J Am Soc Nephrol.

[13] Nakao N, Yoshimura A, Morita H, Takada M, Kayano T, Ideura T (2003). Combination treatment of angiotensin-II receptor blocker and angiotensin-converting-enzyme inhibitor in non-diabetic renal disease (COOPERATE): a randomised controlled trial. Lancet.

[14] Waikar SS, Liu KD, Chertow GM (2008). Diagnosis, epidemiology and outcomes of acute kidney injury. Clin J Am Soc Nephrol.

[15] Waikar SS, Liu KD, Chertow GM (2007). The incidence and prognostic significance of acute kidney injury. Curr Opin Nephrol Hypertens.

[16] Remuzzi G, Bertani T (1998). Pathophysiology of progressive nephropathies. N Engl J Med.

[17] Remuzzi G, Ruggenenti P, Perico N (2002). Chronic renal diseases: renoprotective benefits of renin-angiotensin system inhibition. Ann Intern Med.

[18] Formica RN (2003). CKD series: delaying the progression of chronic Kidney. Hosp Physician.

[19] Meyer TW (2003). Tubular injury in glomerular disease. Kidney Int.

[20] Stenvinkel P, Larsson TE (2013). Chronic kidney disease: a clinical model of premature aging. Am J Kidney Dis.

[21] Elewa U, Sanchez-Niño MD, Martin-Cleary C, Fernandez-Fernandez B, Egido J, Ortiz A (2012). Cardiovascular risk biomarkers in CKD: the inflammation link and the road less traveled. Int Urol Nephrol.

[22] López-Novoa JM, Rodríguez-Peña AB, Ortiz A, Martínez-Salgado C, López Hernández FJ (2011). Etiopathology of chronic tubular, glomerular and renovascular nephropathies: clinical implications. J Transl Med.

[23] Tapia E, Sánchez-Lozada LG, García-Niño WR, García E, Cerecedo A, García-Arroyo FE (2014). Curcumin prevents maleate-induced nephrotoxicity: relation to hemodynamic alterations, oxidative stress, mitochondrial oxygen consumption and activity of respiratory complex I. Free Radic Res.

[24] Appel G (2013). Detecting and controlling diabetic nephropathy: What do we know?. Cleve Clin J Med.

[25] Kher V (2002). End-stage renal disease in developing countries. Kidney Int.

[26] Monedero P, García-Fernández N, Pérez-Valdivieso JR, Vives M, Lavilla J (2011). Acute kidney injury. Rev Esp Anestesiol Reanim.

